# CD40 Gene Silencing Reduces the Progression of Experimental Lupus Nephritis Modulating Local Milieu and Systemic Mechanisms

**DOI:** 10.1371/journal.pone.0065068

**Published:** 2013-06-14

**Authors:** Èlia Ripoll, Ana Merino, Montse Goma, Josep M. Aran, Nuria Bolaños, Laura de Ramon, Immaculada Herrero-Fresneda, Oriol Bestard, Josep M. Cruzado, Josep M. Grinyó, Juan Torras

**Affiliations:** 1 Laboratory of Experimental Nephrology, IDIBELL.Hospital Universitari de Bellvitge, L’Hospitalet, Barcelona, Spain; 2 Pathology Department, Hospital Universitari de Bellvitge, L’Hospitalet, Barcelona, Spain; 3 Medical and Molecular Genetics Center, IDIBELL, L’Hospitalet, Barcelona, Spain; University Heart Center Freiburg, Germany

## Abstract

Lupus nephritis (LN) is an autoimmune disorder in which co-stimulatory signals have been involved. Here we tested a cholesterol-conjugated-anti-CD40-siRNA in dendritic cells (DC) in vitro and in a model of LPS to check its potency and tissue distribution. Then, we report the effects of Chol-siRNA in an experimental model of mice with established lupus nephritis. Our in vitro studies in DC show a 100%intracellular delivery of Chol-siRNA, with a significant reduction in CD40 after LPS stimuli. In vivo in ICR mice, the CD40-mRNA suppressive effects of our Chol-siRNA on renal tissue were remarkably sustained over a 5 days after a single preliminary dose of Chol-siRNA. The intra-peritoneal administration of Chol-siRNA to NZB/WF1 mice resulted in a reduction of anti-DNA antibody titers, and histopathological renal scores as compared to untreated animals. The higher dose of Chol-siRNA prevented the progression of proteinuria as effectively as cyclophosphamide, whereas the lower dose was as effective as CTLA4. Chol-siRNA markedly reduced insterstitialCD3+ and plasma cell infiltrates as well as glomerular deposits of IgG and C3. Circulating soluble CD40 and activated splenic lymphocyte subsets were also strikingly reduced by Chol-siRNA. Our data show the potency of our compound for the therapeutic use of anti-CD40-siRNA in human LN and other autoimmune disorders.

## Introduction

Systemic lupus erythematosus (SLE) is a complex autoimmune disorder affecting multiple organ systems including the kidney, skin, lung, heart, hematopoietic system, and the brain. Type IV glomerulonephritis leading to severe proteinuria, chronic renal failure and end-stage renal disease (ESRD) remains one of the most dreaded complications of SLE and is associated with significant morbidity and mortality [1], [2].

In lupus nephritis insufficient clearance of apoptotic nucleosomes has been postulated as the likely trigger of a T-cell response leading to the formation of autoantibodies which then bind to the glomerular basement membrane and promote inflammation [3], [4]. Renal infiltration by B and T-cells, macrophages, and dendritic cells is a prominent feature of progressive LN leading to renal failure [1]. Some studies have highlighted the importance of T-cells in stimulating the production of autoantibodies by B-cells in SLE [5]. Such stimulatory role by T-cells requires the presence of co-stimulatory signaling dyads, such as CD28/B7 or CD40/CD154, without which B-cells may fail to proliferate or even undergo apoptosis [6], [7].

Among the therapeutic armamentarium available to treat LN, cyclophosphamide (CYP) and steroids can effectively delay the progression of renal disease [8], [9], although failure to achieve remission has been reported in 18–57% of patients. Furthermore, the long term toxicity of CYP and high-dose steroids discourages their chronic use to maintain disease remission [10].

NZB/W F1 mice spontaneously develop an autoimmune disorder which resembles human SLE [11], [12], including the formation of auto-antibodies against multiple epitopes of chromatin and nucleosomes and the presence of haemolytic anemia, proteinuria, and overt nephritis [13], [14], thus providing a suitable experimental model in which to test potential new therapeutic agents. For example, treatment with CTLA4 and a suboptimal dose of CYP has been shown to significantly prolong survival, although without evidence of reduced glomerular immune-complex deposition. Therefore, blocking co-stimulatory signals necessary for T cell activation appears to prevent disease progression in these animals [1], [15], [16]. The co-stimulatory dyad CD40/CD154 (CD40-ligand) has been previously implicated in the pathogenesis of LN and other autoimmune disorders [17], [18].

The administration of LPS is known to dramatically enhance CD40 expression [19], [20]. LPS, a Gram-negative cell wall component recognized by the specific receptor TLR4, is an adjuvant for the adaptive immune response, which up-regulates costimulatory molecules on antigen presenting cells [19]. It has been demonstrated that LPS induces CD40 mRNA and protein expression in both murine and human kidney, heart, brain, small intestine and circulating macrophages [19], [20] thus providing a uniquely challenging experimental model where to test the potency and durability of effect of our specifically designed CD40-siRNA.

RNA-interference (RNAi) is an evolutive innate cell mechanism of post-transcriptional gene silencing, which has been successfully replicated by the administration of synthetic double-stranded small inhibitory RNA (siRNA). Rapid degradation by exo/endonucleases constitutes a serious challenge to the successful intracellular delivery of siRNAs in vivo and their ultimate biological activity. The in vivo potency of a siRNA is thus largely predicated upon sequence specificity and its stability against nucleases [21], [22]. The latter can be achieved through chemical stabilization of the backbone with phosphorothioate (PS) and 2′-O-methyl sugar modifications on the sense and antisense strands [23], [24], or other chemical modifications. The conjugation of cholesterol to the 3′ end of the sense strand by a pyrrolidine linker is also critical to improve cellular uptake Addition of cholesterol prolongs circulating half live but it does not influence its silencing activity [25]. Chemically stabilization or cholesterol conjugation of siRNAs has improved in vitro and in vivo pharmacological and pharmacokinetic properties.

Our group has developed a small inhibitory RNA (siRNA) molecule against murine CD40-mRNAwhich effectively inhibits its translation into CD40 receptor protein [26]. Therefore we here report our experience with a chemically stabilized, cholesterol-conjugated anti-CD40 siRNA (Chol-siRNA) in a model of LPS-induced CD40 up-regulation to assess its potency, distribution and durability of effect following systemic administration. We also focus on the inhibitory effects of our Chol-siRNA CD40 in NZB/W F1 mice with established autoimmune nephritis.

## Methods

### 2.1. CD40-siRNA Properties

The CD40-siRNA sequence used in this study has been previously described by our group (siRNA TNFRSF5-3). It consists of a 21-nucleotides sense strand and antisense strand resulting in a two nucleotides overhang at the 3′ end of the antisense strand (sense 5′-GUGUGUUACGUGCAGUGACUU-3′, antisense 3′-GTCACACAAUGCACGUCACUG-5′).

Cholesterol siRNA (Chol-siRNA): The siRNA was chemically stabilized with partial phosphorothioate backbone and 2′-*O*-methyl sugar modification on the sense and antisense strand, as previously described [25]. And, conjugation of a cholesterol molecule to the 3′ end of the sense strand of the siRNA molecule by means of a pyrrolidine linker was done [25]. Scrambled siRNA (SC) was used as control. The synthesis was carried out by an external manufacturer (Microsynth, Switzerland). To generate siRNAs from RNA single strands, equimolar amounts of complementary sense and antisense strands were mixed and 100% annealed.

Melting point of siRNA molecules was determined using a Jasco V-650 spectrophotometer (Easton, MD, USA) equipped with a Peltier unit. Oligonucleotides strands were annealed at 90°C for 3 minutes and then cooling to 20°C prior to the melting experiment. Samples were heated at linear temperature ramp of 0.5°C/min. Melting temperature was obtained as the maxima of the first derivative.The melting point of the siRNA without modifications is 79°C and for the Chol-siRNA is 71°C.

### 2.2. Bone Marrow Preparation and Dendritic Cell Culture. DC Activation and siRNA Transfection

Femurs and tibiae from ICR mice were left in RPMI-1640medium (Biological Industries, Kibbutz BeitHaemek, Israel). 5×10^6^ cells were grown in 5 ml complete medium: RPMI supplemented with Penicillin (100 U/ml), Streptomycin (100 µg/ml), L-glutamin (2 µM, Reactiva, Almeria, Spain), 10% heat inactivated and filtered FBS and 20 ng/ml GM-CSF (R&D systems, Minneapolis, MN, USA) at 37°C in a humidified atmosphere with 5% CO2. On day 3, 5 and 8 medium was removed and fresh complete medium was added. The percentage of immature dendritic cells was 15% 48% and 90% respectively.

At day 8 two sets of independent experiments were done; one of them to evaluate the transfection of CD40 siRNA molecules: dendritic cells were incubated with nude unmodified siRNA-Cy5.5 or Chol-siRNA-Cy5.5at 2 µM. Continuous cell imaging acquisition was carried out with a confocal microscopy (Leica TCS-SL spectral) for 45 min. The other set aimed to evaluate siRNA in vitro activity: cells were incubated with nude Chol-siRNA or scrambled siRNA at 2 µM. At day 9 DCs were stimulated with 10 ng/ml LPS (serotype O111:B4, Sigma, Madrid, Spain).After 12 h of LPS stimuli, cells were analyzed by flow citometry using a BD FACS CantoII Cytometer and analyzed by FACS DIVA software (BD Biosciences, San Jose, CA, USA). To characterize the phenotype we used the following antibodies: anti-CD11c (clone HL3) and anti-CD11b (clone M1/70), anti CD40 (clone HM40-3) and anti CD80 (clone 16-10A1) anti CD86 (clone GL1) and their respective isotype controls. All antibodies were provided by BD Biosciences, conveniently titrated, mixed together and formulated for optimal staining performance.

### 2.3. Mice, Study Design and Follow Up

After proving that our siRNA effectively internalize into cells, we tested the in vivo effects of Chol-siRNA systemic administration. For this, we used six to eight week old male ICR mice (The Jackson Laboratory, Charles River, Spain). We analyzed CD40-mRNA expression in kidney and liver after i.p. LPS injection (5 µg E.Coli LPS serotype O111:B4, Sigma Aldrich, Madrid, Spain). Animals were euthanized at 4 h and on day 1, 2. In another set of experiments we administered on day 0 a single i.p dose of CD40 Chol-siRNA to assess the effects on kidney CD40-mRNA. Afterwards, at predefined time points (days 0, 1, 3, 5, 7 and 9) an i.p LPS dose was administered and animals sacrificed 4 h later. Scrambed siRNA was used as control.

For lupus nephritis studies, five month old NZB/NZW F1 (The Jackson Laboratory, Charles River, Spain) mice were randomized into five groups and basal studies were done. At sixth month old, treatment was initiated as follows: CYP (n = 9) intraperitoneal CYP, 50 mg/kg every 10 days; CTLA4 (n = 9) intraperitoneal CTLA4 (ORENCIA, abatacept, Bristol Myers Squibb, Spain) 50 µg thrice weekly; siCD40-1w (n = 9) intraperitoneal CD40-siRNA 50 µg once weekly; siCD40-2w (n = 8) intraperitoneal CD40-siRNA (Microsynth, Switzerland) 50 µg twice weekly. Untreated (n = 15) intraperitoneal scranbled siRNA as control, 50 µg twice weekly. Mice were treated for 12 weeks. Body weight was determined twice monthly from the beginning to the end of follow-up. Mice were placed in metabolic cages to collect 24 h urine specimens before the onset of treatment and monthly thereafter. Blood was obtained from the tail vein at monthly intervals. Kidneys were processed for histological and biochemical studies at the end of the study or at death. The spleen was collected, weighted and used to extract splenocytes. Six additional mice from the untreated group were administered I.P and I.V with Chol-SiRNA Cy5.5 labeled to determine organ siRNA biosdistribution in lupus disease.

The experiments were carried out in accordance with current EU legislation on animal experimentation and were approved by “CEEA: *Animal Experimentation Ethic Committee*”, the Institutional Ethics UB Committee for Animal Research. Mice were housed in a constant temperature room with a 12-hour dark/12-hour light cycle, and were given free access to water and a standard laboratory diet.

### 2.4. Proteinuria, Albuminuria and Renal Function

24 h-urinary protein was determined by pyrogallol red and creatinine concentration was determined by Jaffe’s reaction (Olympum Autoanalyzer AU400, Hamburg, Germany) in the Veterinary Clinical Biochemistry Laboratory of Universitat Autonoma de Barcelona. 24 h-urinary albumin was determined using a commercially available ELISA KIT (Active motif, Carlsbad, California, USA) according to the manufacturer’s instructions. The intensity of the fluorescent signal is directly proportional to the albumin concentration in the sample.

### 2.5. RNA Extraction, RT and Gene Expression Analysis: Quantitative Real-time PCR (qPCR)

For molecular studies, the kidney was immediately snap-frozen in liquid nitrogen and stored at −80°C. RNA was extracted from kidney animals with PureLink™ RNA Mini Kit (Invitrogen, Spain) according to the manufacturer’s instructions. All samples had an A _260/280_ ratio <1.8 purity. RNA was stored at −80°C.A total amount of 500 ng of RNA was used to do the reverse transcription using the High-Capacity cDNA reverse Transcription Kit (Applied Biosystems) following the manufacturer’s instructions. Negative controls for reverse transcription were carried out using distilled water.

Tissue expression of immune-inflammatory mediators were quantified by TaqMan real-time PCR (ABI Prism® 7700, Applied Biosystems, Spain) using the comparative CT method (Applied Biosystems, Spain). Taqman gene expression assays used were purchased from Applied Biosystems: CD40 (Mm_00441891_m1), IL1b (Mm_01336189_m1), NLRP3 (Mm_00840904_m1), Apelin (Mm00443562_m1), C3 (Mm01232779_m1), CD40L (Mm00441911_m1), CD55 (Mm00438377_m1), FOXP3 (Mm00475165_m1), IL6 (Mm99999064_m1), IL10 (Mm00439614_m1), MCP1 (Mm00441242_m1), RANTES (Mm01302428_m1) TLR3 (Mm00628112_m1), TLR4 (Mm00445273_m1), TLR9 (Mm00446193_m1), AIM2 (Mm01295719_m1) and Eukaryotic 18S (431941E) as endogenous control. Controls, which were composed of distilled water, were negative for target and reference genes.

### 2.6. 5′RACE

5′ RACE PCR was performed using the 5′ RACE system for rapid amplification of cDNA ends kit (Invitrogen, Madrid, Spain) according to the manufacturer’s instructions. Total (5 µg) mRNA from kidney samples of siRNA treated groups was converted into cDNA using reverse transcriptase and a specific primer (GSP1∶5′GCCGACTGGGCAGGGATGACAGACG3′). To detect cleavage products specific cDNA is then directly amplified by PCR using a specific primer (GSP2∶5′AGCCAGGGATACAGGGCGTGTGC3′) and an adapter primer complementary to the RNA adaptor that targets the tail region. Amplification products were resolved by agarose gel electrophoresis and visualized by ethidium bromide staining.

### 2.7. Plasma ELISA for Anti-DNA Antibodies, CD40, and IDO in Lupus Nephritis

Levels of anti-DNA antibodies were measured, using a commercially available ELISA kit (Alpha Diagnostic International, San Antonio, Texas, USA) according to the manufacturer’s instructions. CD40 protein and indoleamine 2,3-dioxygenase (IDO) was determined in plasma samples at the end of the study using the commercially available ELISA kit RayBio® Mouse CD40/TNFRSF5 (Raybiotech, Norcross, Atlanta, GA, USA) and ELISA kit for IDO (Uscn Life Science, Wuhan, China) according to manufacturer’s instructions.

### 2.8. Serum Cytokine Analysis and Immunoglobulin Isotyping

Serum cytokines and immunoglobulin isotype were quantitatively measured by FACSCanto with different cytometric bead array (CBA) kits according to manufacturer’s instructions: the CBA mouse inflammation kit (IL6, IL10, MCP1, IFNγ, TNF and IL12p70), the CBA mouse Th1/Th2 cytokine kit (IL4, IL5, IFNγ and TNF), and mouse immunoglobulin isotyping kit, all from BD Biosciences (San Jose, CA, USA). Data were acquired and analyzed using BD FCAP software and CBA software (San Jose, CA, USA).

### 2.9. Phenotypic Spleen Population Analysis by Flow Citometry

Spleen was collected in PBS, the splenocytes isolated by Ficoll® (GE Healthcare, Uppsala, Sweden) density gradient and cryo-preserved at −180°C. For quantifying the percentage of different populations, cells were thawed, washed and recovered by standard methods. 2×10^5^splenocytes for tube were incubated in the dark (25 min, RT) with antibodies. Study of populations was performed by using a BD FACS CantoII Cytometer and analyzed by FACS DIVA software (BD Biosciences, San Jose, CA, USA). To characterize the different cell populations we used an anti-CD19 (clone 1D3), anti-CD69 (clone H1.2F3), anti-CD25 (clone PC61), anti-CD3 (clone145-2C11); anti-CD4 (clone RM4-5), anti-CD8 (clone 53-6.7), anti-CD11c (clone HL3), anti-CD11b (clone M1/70),anti-IgD (clone11-26c.2a), anti-CD23 (clone B3B4), anti-CD45R (cloneRA3-6B2), anti mPDCA-1 (clone JF05-1C2.4.1), anti CD5 (clone 53-7.3), and anti CD1d (clone1B1). All antibodies were provided by BD Biosciences and MiltenyBiotec, conveniently titrated, mixed together and formulated for optimal staining performance.

### 2.10. Renal Lupus Histopathology

For histopathological studies, 1–2 mm thick coronal slices of kidney were fixed in 4% formaldehyde and embedded in paraffin. For light microscopy 3–4 µm thick tissue sections were stained with hematoxylin and eosin (H&E) and periodic acid-Schiff.

For determine the extent of renal damage, all renal biopsies were analyzed by two blinded pathologists. Typical glomerular active lesions of lupus nephritis were evaluated: mesangial expansion, endocapillary proliferation, glomerular deposits, extracapillary proliferation and interstitial infiltrates, as well as tubulo-interstitial chronic lesions: tubular atrophy and interstitial fibrosis. Lesions were graded semi-quantitatively using a scoring system from 0 to 3 (0 _ no changes, 1 _ mild, 2 _ moderate, 3 _ severe). Finally, a total histological score (HS) was derived from the sum of all the described items.

Paraffin tissue sections were stained for CD40 (Abcam, Cambridge, UK) and CD3 (Abcam, Cambridge, UK). Sections were de-parafined and hydrated_._ The sections were blocked and immunoperoxidase-labelled using a Vectastain ABC kit and the avidin biotin blocking kit (Vector Laboratories, Burlingame, CA) according to manufacturer’s protocol. Peroxidase conjugated antibodies staining was followed by diaminobenzidine substrate development (Sigma, Madrid, Spain). To quantify CD40 expression a semi quantitative score from 0 to 3 in the different compartments of the kidney (glomeruli, vessels and interstitium) was used. For quantifying CD3 expression at least 15 high-power fields were counted and the mean value was expressed.

### 2.11. Renal Immunofluorescence in Biodistribution Studies and Lupus Nephritis

Slices of kidney were fixed in 4% paraformaldehyde, embedded in Tissue Tec OCT compound (Sakura, Netherlands) and stored at −80°. Five-µm cryostat sections were used for confocal microscopy to quantify the mean fluorescence intensity of siRNA-Cy5.5 or Cy5.5 fluorochrome. At least 10 HP fields from each organ were counted.

For analysis of IgG and C3 deposition, fluorescent staining of cryo-sections were used. Sections were directly stained with FITC-conjugated goat anti-mouse IgG (Sigma-Aldrich, Spain), and FITC conjugated C3 (Nordic Immunology, Netherlands). For analysis of C3 and IgG deposition at least 10 glomeruli were visualized and photographed with an immunofluorescence confocal microscope (Leica TCS-SL spectral). Fluorescence was quantified with Leica software and expressed as mean fluorescence intensity. For the localization and quantification of interstitial plasma cells paraffin sections were stained with FITC-conjugated goat anti-mouse IgG (Sigma-Aldrich, Spain) and rabbit anti mouse collagen-IV (Chemicon international, Temecula, CA). Sections were incubated with primary antibodies Col-IV and IgG in goat serum overnight. Staining was visualized with a secondary chicken anti goat Alexa 488 and a goat anti rabbit Alexa 546 (Invitrogen, Madrid, Spain). For quantification of plasma cells at confocal microscopy, a semi quantitative score of distribution from 0 to 3 was used.

### 2.12. Statistical Analysis

Overall survival was analyzed by the Kaplan-Meier method. One-way analysis of variance (ANOVA) with *post hoc* tests was performed to compare proteinuria, anti-dsDNA antibodies, gene expression, and spleen population, throughout the follow up. To compare histological data, the non-parametric Kruskal-Wallis test was used. P value <0.05 was considered significant. Data are expressed as mean±SEM.

## Results

### 3.1. DCs were Efficiently Transfected by siRNA. Chol-siRNA Inhibited CD40 Expression in DCs

**Figure 1:**
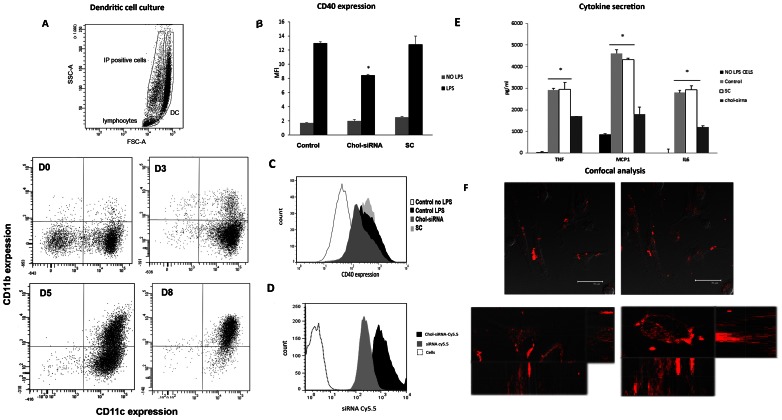
Representative flow cytometry analysis of DC culture. A) DC culture development. Lymphocytes (CD3+) and death cells (IP positive) were discarded. DCs were backgated using a FSC-A/SSC-A dot plot. Subsequently, CD11c and CD11b expression were assessed in DC population using CD11c APC/CD11bPE dot plot. D0, D3, D5 and D8 = day 0, 3, 5 and 8 respectively B) CD40 expression of DC with and without LPS stimuli and transfected with Chol-siRNA or SC siRNA were analyzed by flow cytometry (MFI). *p<0.05 vs control and scrambled. C) Representative histograms for each group of treatment. D) Representative histograms of siRNA internalization analyzed by flow citometry (MFI). E) DC cytokine secretion after LPS stimulus and Chol-siRNA effect. F) Chol-siRNA Cy5.5 internalization in DCs is shown at time 0 and after 45 min of incubation (upper left and right photomicrograph x630, respectively). Middle and bottom photomicrographs: Z-scan image of live DC cells where we can observe siRNA cytoplasm localization and confocal projections of the cell to the xz and zy planes (sideviews). Data are expressed as mean± SEM of four separate experiments.

The effective transfection of siRNA into DCs was examined using Cy5.5 labeled siRNAs. siRNA molecules were efficiently delivered into the cell(100%)(data not shown);and as seen in fig. 1,Chol-siRNA-Cy5.5 had a significantly higher MFI (mean fluorescence intensity) than unmodified siRNA. To evaluate cell viability after gene transfection, DCs were stained with propidium iodide 12 h after LPS stimulus. Cell viability was not affected due to the siRNA transfection.

After 45 min of DC incubation with Chol-siRNA, the molecule was totally located in the cytoplasm of the cell (Fig. 1). This was also confirmed by Z-scan imaging of DC. These results indicate that Chol-siRNA is cell permeable and could be delivered efficiently into the cells.

To investigate the effect of siRNA transfection on the expression of CD40 gene, we used LPS to induce DC maturation and CD40 overexpression. DC maturation was confirmed by flow citometry 12 h after LPS stimulus analyzing the increase in CD40, CD80 and CD86 cell surface expression (basal vs stimulated MFI),CD40∶1.7±0.0 vs 12.9±0.2; CD80∶2.8±0.0 vs 5.2±0.0; CD86∶1.2±0.0 vs 6.3±0.0). Results show that the unstimulated DC, independently of the siRNA used, did not change CD40 expression. When cells were exposed to LPS, only DC treated with Chol-siRNA decreased 35% the CD40 expression respect to the control and SC group. (Fig. 1). CD80 and CD86 expression showed a non significant reduction (results not shown). LPS stimulus induced a clear release of TNF, MCP1 and IL6 in supernatants. Anti CD40 siRNA administration induced a significant reduction in all those cytokines (Fig. 1).

### 3.2. in vivo Study of the Inhibitory Effects of Chol-siRNA on Renal CD40-mRNA Expression after LPS Injection

**Figure 2:**
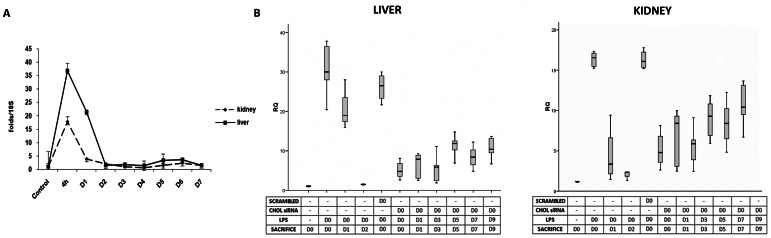
In vivo CD40 expression after LPS stimulus and Chol-siRNA. A) ) Kinetic of CD40 expression in liver and kidney after LPS injection, a peak of CD40 expression at 4 h was seen. There were no differences using scrambled siRNA. B) Pharmacodynamic evaluation of Chol-siRNA. Mice receiving a single dose of Chol-siRNA at day 0 and a single injection of LPS at pre determined time points showed reduction on liver and kidney CD40 gene expression. Data are expressed as mean± SEM of four separate experiments.

Renal and hepatic CD40-mRNA expression was nearly 20 and 35-fold higher than control, respectively, 4 hours after LPS injection (Fig. 2). Renal CD40-mRNA expression returned towards control values within 24 hours after LPS injection, whereas it remained elevated for approximately 48 hours in the liver. After a single initial Chol-siRNA administration and LPS injection on predetermined time-points, renal and hepatic CD40-mRNA expression was reduced by approximately 65% and 60& respectively for 3 days compared to both controls (no siRNA and SC group), and persisted for up to 5 days (Fig. 2). As a control, TLR4 mRNA was activated by LPS injection. Chol-siRNA reduced over-expression of TLR4 LPS-induced (30,1 and 12,1 folds respectively, not shown).

### 3.3. Animal Survival, Proteinuria and Albuminuria

**Figure 3:**
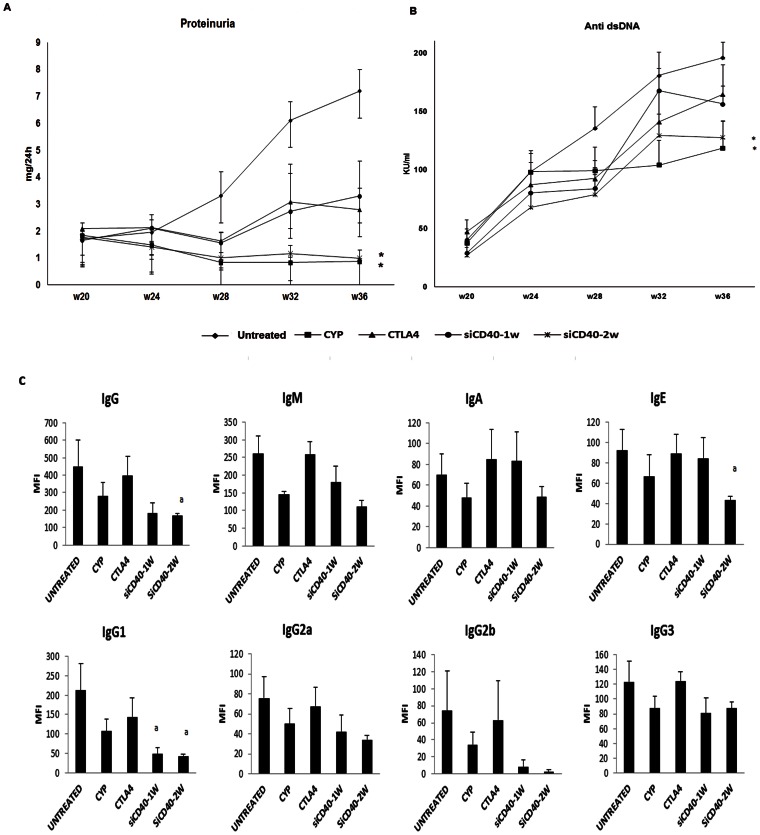
Proteinuria, anti dsDNA antibodies and Immunoglobulin isotype. A) 24 h-Proteinuria increased progressively in non treated mice to levels of heavy proteinuria. Treatment with CYP or siRNA anti CD40 twice per week induced a progressive reduction of proteinuria to levels almost physiological. B) Anti dsDNA antibodies increased progressively in non treated mice; siRNA anti CD40 twice per week reduced this production more effectively than CTLA4 or siCD40-1w group. C) Circulating immunoglobulin subclasses characterization. CD40 blockade with Chol-siRNA reduced total IgG and all IgG fractions. Data are expressed as mean±SEM of four separate experiments, p<0.05 a vs untreated.

Cumulative survival analyzed by Kaplan-Meier method was 100% for the CYP, CTLA4 and CD40-2w groups; 88% for the CD40-siRNA-1w group, and 73% for the untreated group at the end of the follow up. Proteinuria (Fig. 3a) and albuminuria (not shown) increased progressively in untreated mice to levels of heavy proteinuria (around 300 mg/kg) though the mortality, proteinuria levels continued to increase. The CTLA4 and siCD40-1w groups showed no increase in proteinuria or albuminuria until week 28, but to a lesser extent than the untreated group. Mice treated with CYP or siCD40-2w showed no increase in either parameter, or even a mild reduction in proteinuria over time to levels almost physiological.

### 3.4. Anti dsDNA Antibodies in Lupus Nephritis. Immunoglobulin Isotype

IgG anti-dsDNA antibody levels increased steadily in untreated group (Fig. 3b). As expected, CYP slowed down the production of antibodies. Chol-siRNA produced a dose dependent reduction of antibodies. Treatment of mice with siRNA once per week partially reduced antibodies, similarly as CTLA4 treatment. The levels in the CYP and siCD40-2w groups were significantly lower from untreated at week 36 (p<0.05).

Immunoglobulin subclasses characterization showed that IgG was the most frequent isotype in serum (Fig. 3c). Anti dsDNA antibody, in particular of the IgG isotype are considered a hallmark of LES and correlates with disease activity. CD40 blockade with chol-siRNA, either one or twice per week, reduced total IgG and all IgG fractions. Levels of IgM, IgA and IgE were reduced in siCD40-2w group, which was significant for IgE (Fig. 3c).

### 3.5. Renal Structural Effects of CD40 Chol-siRNA in Lupus Nephritis

**Figure 4:**
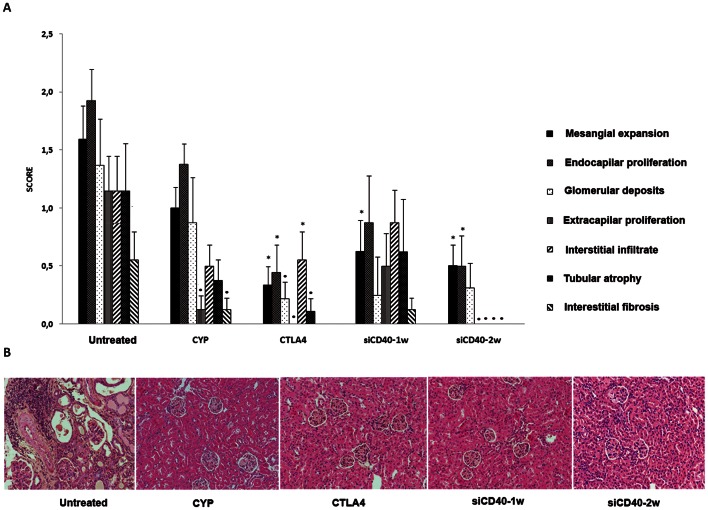
Renal histopathology. A) Costimulatory blockade reduced the elementary histological lesions of lupus nephritis. B) Representative photomicrograph (x200) of renal histology for each group. Data are expressed as mean±SEM *p<0.05 vs untreated, p<0.01 vs untreated.

Renal histological analysis was performed in all surviving animals at the end of the study (Fig. 4).Untreated mice kidneys showed lesions consistent with proliferative lupus nephritis, including severe glomerulonephrits, interstitial inflammation, and widespread proteinaceous tubular casts as expected from their severe proteinuria. Histological lesions were less severe in all treated groups. Mean histological scores were as follow: Untreated = 8±1.5; CYP = 4.3±1.1; CTLA4 = 1.6±0.7; siCD40-1w = 3.8±1.5; and siCD40-2w = 1.6±0.6. (p = 0.011) being siCD40-2w and CTLA4 groups statistically significant respect to control group. With regards to individual histological lesions, the siCD40-2w group was remarkable for the absence of extracapillary proliferation, interstitial infiltrates, tubular atrophy, or interstitial fibrosis. This effect was clearly better than mice receiving siRNA once a week.

### 3.6 in vivo Determination of CD40 Chol-siRNA Renal Release and Effective CD40 mRNA Cleavage in Lupus Nephritis

**Figure 5:**
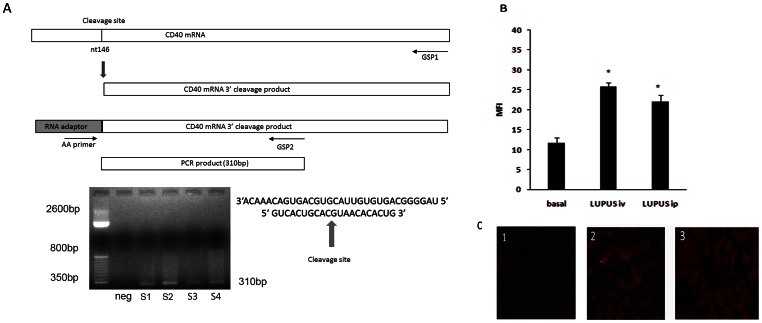
siRNA-mediated cleavage of CD40 mRNA in vivo. Confocal fluorescence and brightfield images of siRNA internalization in DC. A) Schematic representation of the CD40 mRNA illustrating siRNA cleavage sites and RACE strategy to detect cleavage product. Agarose gel of RACE–PCR amplification showing specific cleavage products in different lupus nephritis samples (S1 and S2), and in vitro DC culture (S3 and S4). B) Quantification of renal internalization of Chol-siRNA administered iv/ip in lupus mice. C) Representative kidney photomicrographs (x400) of 1- Basal auto fluorescence, 2- iv administration, 3- ip administration. Data are expressed as mean±SEM of four separate experiments.*p<0.05 vs basal.

Additional lupus mice that received Chol-siRNA Cy5.5 labeled one hour before the sacrifice, showed an intense fluorescence in tubular cells (Fig. 5). Thus in the kidney the fluorescence intensity doubled the basal value.

To prove that the in vivo activity was due to siRNA-directed cleavage, we characterized specific CD40 mRNA cleavage products using a 5′-RACE technique. Total RNA from mice was isolated from renal tissue, and then PCR and agarose gel were used to reveal fragments of the predicted length in those animals with lupus nephritis receiving chol-siRNA (Fig. 5). This demonstrates that cleavage occurred at the predicted position for the siRNA duplex, ten nucleotides downstream of the 5′ end of the siRNA antisense strand. To further assess the cleavage of siRNA in immune cells we performed the 5′-RACE technique on dendritic cells stimulated by LPS and treated with siRNA anti CD40 and a correct cleavage was seen (Fig. 5).

### 3.7. CD40 Silencing Reduces IgG and C3 Glomerular Deposits

**Figure 6:**
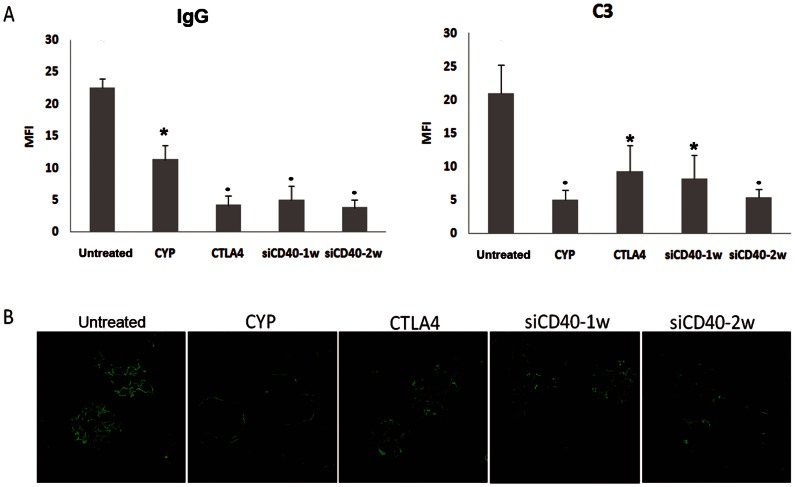
Immunohistochemical analysis for renal IgG and C3. A) Deposits of renal IgG and C3 were quantified with confocal microscopy (MFI). All treatments reduced glomerular deposits. B) Representative photomicrographs of C3 deposits (x630) for each group. Data are expressed as mean±SEM. *p<0.05 vs untreated, · p<0.01 vs untreated.

IgG glomerularimmunostaining was reduced by all treatments as compared to untreated untreated animals (Fig. 6a).Both CTLA4 and siCD40-2w produced similar reductions in IgG immune-fluorescence scores, which were lower than in the CYP group. C3 deposits were also reduced in all treatment groups, although in this case scores in the CYP and siCD40-2w were lower than in the CTLA4 and siCD40-1w group. (Fig. 6a).

### 3.8. Renal Gene Expression and CD40 Protein Analysis After Silencing in Lupus Nephritis

The analysis of kidney gene expression (Table 1) showed a statistically significant reduction in CD40 expression with CYP and siCD40-2was compared to the untreated and CTLA4 groups. All treatment groups produced a significant reduction in CD40L compared to untreated (except siCD40-1w).

**Table 1:** Kidney gene expression.

Fold/18S	CD40	CD40L	C3	CD55	TLR3	TLR4	TLR9	FOXP3
**UNTREATED**	1,37±0,1	5,3±1,4	47,3±14,1	1,02±0,08	1,7±0,1	2,9±0,4^b^	8,2±1,1^b^	34,8±6,4
**CYP**	0,55±0,1 ^a,c^	1,7±0,3^a^	10,5±0,9^a^	0,74±0,04^a^	1,4±0,1	1,4±0,1	1,7±0,1	7,2±1,3^a^
**CTLA4**	1,17±0,2	2,8±0,5^a^	18,8±4,8^a^	0,79±0,08^a^	1,4±0,1	1,9±0,2^a^	5,1±1,1^b^	20,3±5,6^a, b^
**siCD40-1w**	1±0,3	3,8±0,3^b^	38,8±14,6^b^	0,81±0,04^a^	1,6±0,1	2,5±0,3^b^	7,1±0,9^b^	24,6±2,6^b^
**siCD40-2w**	0,6±0,2^a,c^	2,2±0,4^a^	16,3±3^a^	0,62±0,07^a^	1,7±0,1	2,5±0,3^b^	7,4±1,6^b^	20,9±3,8^a, b^
**P**	0,0251	0,004	0,03	0,01	0,17	0,02	0,0009	0,0034
**Fold/18S**	**IL10**	**IL6**	**MCP1**	**RANTES**	**AIM2**	**NLRP3**	**IL1b**	**APELIN**
**UNTREATED**	90,9±20,5	9,3±3,6	9,3±1,9^b^	9,3±1,3^b^	11,3±2,2	2,7±0,4	11,9±2,1	6,2±0,9^d^
**CYP**	6,1±2,1^a^	0,7±0,15^a^	2±0,2	1,9±0,3	1,4±0,2^a^	0,6±0,2	1,8±0,3	6,3±0,4^d^
**CTLA4**	34,8±9,3^a^	3,3±0,9^a^	5,9±1,7	7,4±2,5	4,6±1,1^a^	2,08±0,3	6,5±1,7	6,2±0,4^d^
**siCD40-1w**	44,1±9,6^a, b^	6,1±2,5^b^	7,3±1,9^b^	9,6±3^b^	7,9±1,9^a,b^	1,5±0,4	8,8±1,6	5,5±0,4^d^
**siCD40-2w**	35,45±12,9^a^	1,8±0,3^a^	6,5±1,2^b^	7,8±1,7	4,9±1,0^a^	1,3±0,7	1,9±1,0	12,1±1,1
**P**	0,0005	0,024	0,039	0,09	0,0046	0,019	0,0007	0,0001

Our Chol-siRNA specifically knocked-down CD40 expression. Complement activation, inflammasome and innate immunity gene expression was reduced. a vs control,n = 9 per group. One-way analysis of variance (ANOVA) with *post hoc* tests was performed to compare groups. a vs control, b vs CYP, c vs CTLA4, d vs siCD40-2w. p<0,05.

C3 gene expression, as a manifestation of local complement synthesis, was significantly reduced by in all treatment groups except siCD40-1w and mirrored the results of glomerular C3 deposition analysis. There was no evidence of activation of TLR3, TLR4 and TLR9 expression in any treatment group as compared to untreated values. Pro-inflammatory cytokine gene expression, such as IL6, was again significantly reduced in all treatment groups except siCD40-1w. Gene expression of the inflammasome components AIM2, NALP3 and IL1b was significantly reduced in all treatment groups.AIM2 is an inflammasome component known to be a sensor for dsDNA and an activator of caspase 1.Gene expression corresponding to the immune-modulatory mediators FOXP3 and IL10was significantly reduced in all treatment groups, particularly CYP. However siCD40-2w did not reduce its gene expression as strongly as CYP. siCD40-2w was the only treatment which up-regulated the expression of apelin, as previously shown by our group [27].

**Figure 7:**
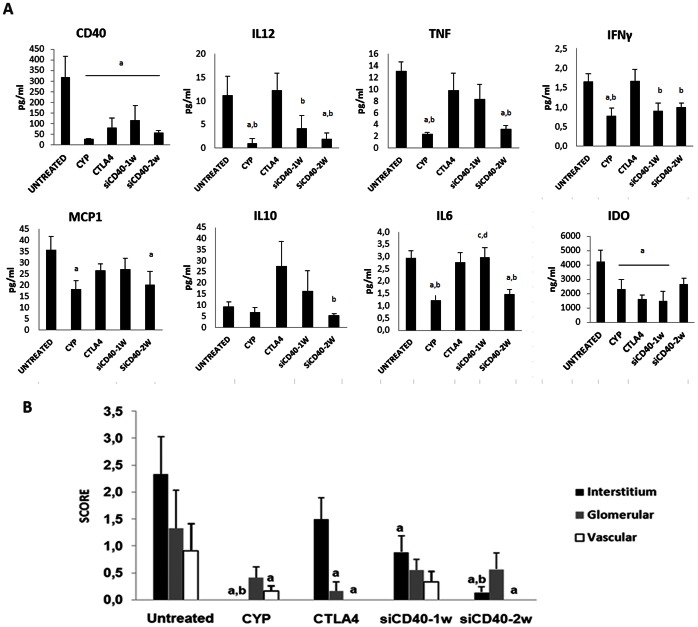
Systemic circulating inflammatory cytokines and local CD40 immunostaining. A) Lupus nephritis promoted over-expression of CD40 protein in serum, this immune modulatory protein was reduced in all therapies. Lupus nephritis also induced the increase in other inflammatory cytokines, treatment with siRNA anti CD40 reduced some of them. B) Immune localization and quantification of CD40 protein in different kidney compartments, Chol-siRNA reduced CD40 protein, especially in interstitial cells and vessels. Data are expressed as mean±SEM. a p<0.05 vs untreated; b vs CTLA4, c vs CYP and d vs siCD40-2w.

The presence of CD40 protein in various renal tissue compartments was quantified by immune-staining (Fig. 7b). Significant reductions were observed in the interstitial, glomerular, and vascular compartments in all treatment groups as compared to untreated, where high amounts were present in all three compartments. siCD40-2w especially reduced CD40 protein in interstitial cells and vessels.

### 3.9. Systemic Circulating Cytokines in Lupus Nephritis

Lupus nephritis induced an increase in circulating CD40 protein (319±98 versus76±12pg/ml in healthy mice).Circulating levels ofCD40 protein (Fig. 7a) were significantly reduced (P = 0.019) in all treatment groups as compared to the high levels observed in the untreated group.

Also, serum levels of pathogenic pro-inflammatory cytokines, such as IL12, TNF, IFNγ, MCP1 and IL6, were increased compared to healthy animals (data not shown). A clear reduction in all of them was seen by siRNA anti CD40 twice per week treatment (Fig. 7a).

Circulating levels of the adaptive immune-modulatory IDO protein (Fig. 7a) were also significantly reduced (P = 0.035) in all treatment groups except siCD40-2w where the difference from untreated did not reach statistical significance.

### 3.10. Analysis of Splenic Lymphoid Cell Subsets in Lupus Nephritis

Spleen weight was higher than normal in untreated group animals and was significantly reduced in the CYP and siCD40-2w treatment groups (data not shown).

Phenotypic characterization of splenic lymphocytes by flow cytometry (Table 2) showed a significant reduction in CD3+ cells in all treatment groups, whereas activated CD3 subpopulations, as well as the CD4+/CD8+ ratio, where reduced by CYP and siCD40-2w (data not shown). Spleen Treg cells (CD3+CD4+CD25+) showed a non significant reduction in CYP group, with no differences between the other groups (Table 2).

**Table 2:** Immunomodulation of spleen cell population (%).

%GROUP	CD3+	Treg	CD11c+	pDC	cDC CD8+	cDC CD8-
**UNTREATED**	42,6±2,1	9,16±1	10,1±1,1	15,4±1,9^b^	12,3±1,3	38,8±2,6
**CYP**	34,2±1,6^a^	5,32±1,2	11,8±0,7	26,8±2,5	12,3±0,8	44,1±1,1
**CTLA4**	31,1±1,2^a^	9,48±1,9	10,6±0,6	15,8±2,4^b^	7,1±0,6^a^	37,4±3,4
**siCD40-1w**	32,1±1,9^a^	10,48±0,8	11,8±1,7	19,4±0,9^b^	8,8±1,2^a^	36,8±0,8
**siCD40-2w**	31,1±1,5^a^	8,88±1,9	9,4±0,6	13,8±1,8^b^	8,4±0,4^a^	40,8±2
**P**	0,0011	NS	NS	0,002	0,006	NS
**%**	**CD19+**	**CD19+CD69+**	**CD19+CD25+CD69+**	**Bregs**	**B1**	**B2**
**UNTREATED**	39,5±3,7	10,6±2,5	3,2±0,7	6,6±0,9	11,2±0,9	60,9±1,7
**CYP**	31,7±2,1	7,2±1,1	2,7±0,5	12,4±1,3^a^	15,4±2,1	48,6±3,0
**CTLA4**	43,2±0,8^b^	7,2±0,4	2,3±0,2	5,78±1,9	11,8±1,6	53,3±7,2
**siCD40-1w**	37,8±4,6	6,2±1,1	1,6±0,3^a^	5,9±1,8	18,2±3,4^a^	48,8±7,3
**siCD40-2w**	39,7±2,1	4,1±1,1^a^	1,1±0,3^a,b^	10,3±1,8^a^	18,9±3^a^	51,9±4,3
**P**	NS	0,045	0,05	0,022	0,07	NS

The percentage of B cell was diminished with CYP treatment, CD40 silencing reduced early B cell activation but not overall B cell population. n = 9 per group. One-way analysis of variance (ANOVA) with *post hoc* tests was performed to compare groups. a vs control, b vs CYP, c vs CTLA4, p<0,05.

Plasmacytoid DCs (pDC) (CD11c+CD11b-B220) were significantly augmented in CYP group. Conventional DC (cDC) are usually divided into CD8+ or CD8-; in our study cDC CD8+ were significantly reduced in those groups with costimulation blockade. There were no changes in cDC CD8- between different groups (Table 2).

With regards to CD19+ B-cells, only CYP caused areduction whereas early B-cell activation markers(CD19+CD25+CD69+ & CD19+CD69+) showed a significant reduction only in both CD40-siRNA groups. This suggests that, while CYP can reduce overall B-cell populations, it does not affect their activation process whereas costimulation blockade reduces early B cell activation but not overall B cell population.

Breg cells (CD19+CD5^high^ CD1d^high^) were significantly augmented in CYP and siCD40-2w groups. Interestingly, there was an inverse correlation between the percentage of spleen Breg cells and the levels of circulating anti dsDNA antibodies (p = 0.018, r = 0.5) Innate B1 cells (CD19+B220^lo^ CD23-IgD^lo^) showed a significant increase in groups treated with siRNA anti CD40, either once or twice per week. There was no differences respect to the percentage of conventional B2 cells (CD19+B220^high^CD23^high^IgD^high^) between different groups. Plasma cells (CD19+B220-CD9+CD23-IgD-) were significantly increased in CYP group, but no in siCD40 treated groups (data not shown).

### 3.11. Characterization of Cell Infiltrates and Localization of IgG-secreting Plasma Cells in the Kidney

**Figure 8:**
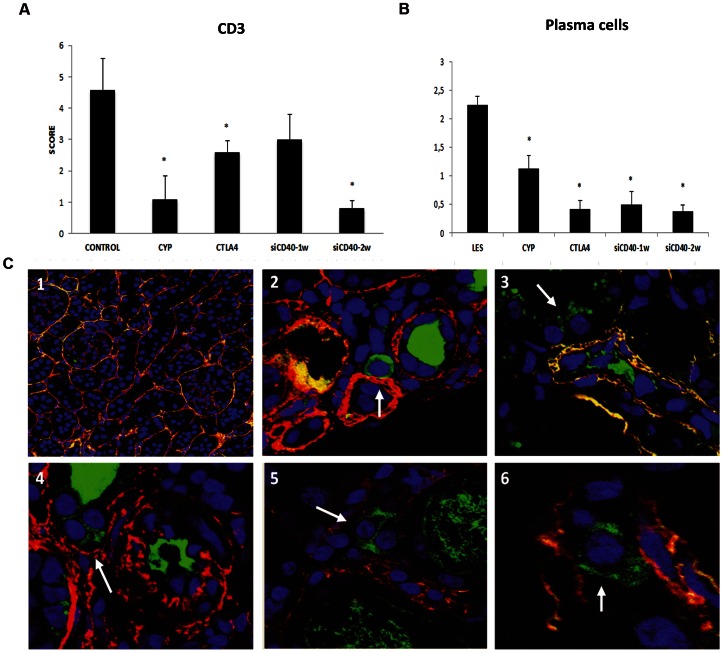
Kidney cell infiltrate characterization and plasma cell localization. A) CD3 presence was semi quantitatively graded B) Plasma cells were semi quantitatively graded and localized in the tubule-interstitium of renal cortex. C) 1.Representative photomicrograph (x400) of siCD40-2 group. Note the absence of plasma cells. 2–6. Photomicrographs (x630) of plasma cell localization in untreated group (arrows). Data are expressed as mean±SEM. *p<0.05 vs untreated.

Infiltrating CD3+ cells in the tubule-interstitium space were significantly reduced in all treatment groups except siCD40-1w (Fig. 8a); more particularly in the CYP and siCD40-2w groups where scores were significantly lower than in the CTLA4 and siCD40-1w groups.

The presence of anti-dsDNA antibody secreting plasma cells in the kidneys of patients with lupus nephritis has been previously reported [28]. Thus, we performed a double immune-staining of renal tissue with anti-collagen-IV (red in Fig. 8c) and anti-IgG (green in Fig. 8c). In untreated animals, plasma cells were mainly observed in the tubule-interstitium of the renal cortex where dendritic cells, macrophages, and other inflammatory cells are commonly found [28].A significant reduction in IgG immune-fluorescence was observed in all treatment groups, particularly in the CTLA4 and both CD40-siRNA groups (Fig. 8b).

## Discussion

Our data from a well-recognized experimental murine model of autoimmune nephritis shows that interfering with the expression of the co-stimulatory molecule CD40 with a selective siRNA results in improved animal survival and reduces the progression of the disease process, as illustrated by the absence of progressive proteinuria. These effects in mice were accompanied by significantly reduced circulating anti-dsDNA antibody titers, a hallmark of the disease, and renal histo-pathological lesions to a degree which was surprisingly comparable to those obtained with cyclophosphamide. Our data also show highly concordant reductions in intra-renal cellular (T-cells, plasma cells), and non-cellular (IgG and C3 deposits) inflammatory components, as well as circulating CD40, IFN, TNF, IL6, MCP1, IL12 factors with obvious pathogenesis implications.

First of all we chose LPS-induced CD40-mRNA up-regulation as a particularly challenging model where to test the potency and pharmacokinetics/dynamics of our Chol-siRNA. There, our data show that LPS induces large increases in CD40-mRNA expression in both liver and kidney tissue which can be significantly reduced by a single dose of Chol-siRNA. Its suppressive effect is remarkably long-lasting in both liver and kidney, particularly so in the latter where it remains unabated for the first 3 days. Interestingly, the biodistribution fluorescence studies showed an excellent internalization into those parenchymas. There is no discernible difference with the I.P. and I.V route with regards to its persistence over time.

The CD40-CD154 co-stimulatory dyad plays a central role in the development of immune-inflammatory disease processes [6], [29]. Disruption of CD40 signaling offers the potential of being therapeutically useful in autoimmune inflammatory disorders or in the prevention of allograft rejection [26]. Blocking monoclonal antibodies against CD40L have reached the clinical testing stage but their development has been halted due to athero-thrombotic complications in study subjects [17], [18]. Cyclophosphamide has been in use for decades to treat SLE and LN, but its short and long term toxicity and relatively low effectiveness in preventing disease relapse [10] has made its use progressively less prevalent, as more than 50% of patients with lupus nephritis relapse within two years after cessation of therapy. Besides cyclophosphamide, we included in our experimental design CTLA4, which was only as powerful as a single weekly dose of CD40-siRNA in halting the progression of proteinuria, clearly a sub-therapeutic dose when compared to the effects of the bi-weekly CD40-siRNA dosing regimen.

SLE is characterized by excessive activation of both B and T lymphocytes, as well as abnormalities in B-cell activation, signaling, and migration [30], [31]. In this study we found a decrease in the frequency of spleen B cells in mice treated with CYP compared with untreated mice. Costimulatory blockade with our siRNA anti CD40 did not modify the percentage of B cells, but in contrast, it prevented early activation of spleen B and T cells. It could be speculated that reduction of dsDNA antibodies in siRNA treated mice is probably due to the modulation of B-cell rather than an antiproliferative effect. In concordance, Breg and B1 cells were clearly increased in animals receiving CD40 siRNA twice per week. The clear-cut inverse correlation between Breg and anti dsDNA antibodies titters also argues in favour of this. Breg cells have a modulatory function by suppressing IFNγ and TNF from T cell, and releasing several regulatory cytokines. Similar to Breg, B1 cells exert immunosuppressive effect [32]. We did not find differences in Treg population in our study thus it may be argued that in this model the regulatory arm should be more related to Bregs than Tregs.

Any of studied treatments induced differences in overall CD11c+ population, but siRNA anti CD40 treatment twice per week was able to significantly reduce CD40 expression in those cells. The cDC CD8+ are thought to be important maintaining self-tolerance and, in vivo, they secrete high amounts of IL12 and IFNγ [31], inducing a Th1 response. On the other part, cDC CD8- mainly induce Th2 response and secrete low levels of IL12. Of note, in our study no differences between cDC CD8- were found but a significant reduction of cDC CD8+ in both siRNA groups was seen.

Effective CD40 gene silencing appears also to produce systemic effects as is the reduction of immune system response, as illustrated by the decrease in circulating CD40, IL12, TNF, IFNγ, MCP1, IL6 proteins. CD40-siRNA did not increase the expression of the immune-modulatory mediator neither local nor circulating IL10, which was locally reduced by cyclophosphamide. The role of IL10 in LES is controversial, it has been considered as an anti-inflammatory mediator, but some studies demonstrate that the administration of IL10 accelerates the onset of renal disease, high serum levels of IL10 are found in patients with SLE and correlates with the disease activity [33]–[35].

Recently, antibody secreting cells were found within the inflamed kidneys in experimental as well as human SLE [28], [36], [37]. In our study we confirmed the presence of plasma cell within the renal interstitium of lupus non treated mice; their presence was diminished in siRNA CD40 treated groups. These data may suggest that our siRNA exerts an intense local effect on plasma cell nidation, added to the systemic benefit on B-cell activation.

The complement system plays a dual role in the pathogenesis of LN [38], [39]. It is known that kidney contributes to the circulating pool of C3, approximately for 9% of the total circulating protein [40]. In our study siRNA anti CD40 treated mice showed an important down regulation of glomerular C3 deposits. In addition the reduction of C3 gene expression in the kidney in siRNA twice per week informs that CD40 gene silencing also reduces the local synthesis of complement. Apart from complement modulation, infiltrating T-cells were reduced as an effect of CD40 silencing thus suggesting a modulation of subsequent local inflammatory response.

Gene expression analysis corroborated the reduced CD40 and inflammatory cytokines in renal tissue without evidence of toll-like receptor activation. This is a pathway for innate immune system activation of potential concern with the use of siRNAs, particularly with regards to TLR3[41]–[43]. In our study, the expression of TLR9 was expectedly increased in non treated animals, and was dramatically reduced by cyclophosphamide but not CTLA4 or CD40-siRNA, suggesting that, in contrast to cyclophosphamide, co-stimulatory blockade does not impair innate immunity through this pathway.

In conclusion, our data strongly supports the potential therapeutic effects of selective CD40 blockade as a powerful form of immune deactivation in the inflamed kidney of animals with spontaneous autoimmune nephritis akin to human lupus nephritis, particularly when treatment is instituted during the early phases of the disease. It remains to be seen whether disease remission could be achieved through CD40 RNA-interference initiated during the established phase of the disease, since patients with lupus nephritis often present with established proteinuria and severe renal inflammation.

## References

[1] Bagavant H, Fu SM (2009) Pathogenesis of kidney disease in systemic lupus erythematosus. Curr Opin Rheumatol 21: 489–494. 19584729 10.1097/BOR.0b013e32832efff1 PMC2841319

[2] Robson MG, Walport MJ (2001) Pathogenesis of systemic lupus erythematosus (SLE). Clin Exp Allergy 31: 678–685. 11422126 10.1046/j.1365-2222.2001.01147.x

[3] Berden JH (2003) Lupus nephritis: consequence of disturbed removal of apoptotic cells? Neth J Med 61: 233–238. 14628957

[4] Davidson A, Aranow C (2010) Lupus nephritis: lessons from murine models. Nat Rev Rheumatol 6: 13–20. 19949431 10.1038/nrrheum.2009.240 PMC4120882

[5] Connolly K, Roubinian JR, Wofsy D (1992) Development of murine lupus in CD4-depleted NZB/NZW mice. Sustained inhibition of residual CD4+ T cells is required to suppress autoimmunity. J Immunol 149: 3083–3088. 1357036

[6] Grewal IS, Flavell RA (1998) CD40 and CD154 in cell-mediated immunity. Annu Rev Immunol 16: 111–135. 9597126 10.1146/annurev.immunol.16.1.111

[7] Davidson A, Wang X, Mihara M, Ramanujam M, Huang W, et al (2003) Co-stimulatory blockade in the treatment of murine systemic lupus erythematosus (SLE). Ann N Y Acad Sci 987: 188–198. 12727639 10.1111/j.1749-6632.2003.tb06048.x

[8] Russell PJ, Hicks JD, Burnet FM (1966) Cyclophosphamide treatment of kidney disease in (NZB x NZW) F1 mice. Lancet 1: 1280–1284. 4160875

[9] Cunnane G, Chan OT, Cassafer G, Brindis S, Kaufman E, et al (2004) Prevention of renal damage in murine lupus nephritis by CTLA-4Ig and cyclophosphamide. Arthritis Rheum 50: 1539–1548. 15146424 10.1002/art.20147

[10] Ioannidis JP, Boki KA, Katsorida ME, Drosos AA, Skopouli FN, et al (2000) Remission, relapse, and re-remission of proliferative lupus nephritis treated with cyclophosphamide. Kidney Int 57: 258–264. 10620207 10.1046/j.1523-1755.2000.00832.x

[11] Sang A, Yin Y, Zheng YY, Morel L (2012) Animal models of molecular pathology systemic lupus erythematosus. Prog Mol Biol Transl Sci 105: 321–370. 22137436 10.1016/B978-0-12-394596-9.00010-X

[12] Huston DP, Steinberg AD (1979) Animal models of human systemic lupus erythematosus. Yale J Biol Med 52: 289–305. 380186 PMC2595466

[13] Borchers A, Ansari AA, Hsu T, Kono DH, Gershwin ME (2000) The pathogenesis of autoimmunity in New Zealand mice. Semin Arthritis Rheum 29: 385–399. 10924025 10.1053/sarh.2000.7173

[14] Hahn BH (2001) Lessons in lupus: the mighty mouse. Lupus 10: 589–593. 11678444 10.1191/096120301682430140

[15] Schiffer L, Sinha J, Wang X, Huang W, von Gersdorff G, et al (2003) Short term administration of costimulatory blockade and cyclophosphamide induces remission of systemic lupus erythematosus nephritis in NZB/W F1 mice by a mechanism downstream of renal immune complex deposition. J Immunol 171: 489–497. 12817034 10.4049/jimmunol.171.1.489

[16] Wang X, Huang W, Mihara M, Sinha J, Davidson A (2002) Mechanism of action of combined short-term CTLA4Ig and anti-CD40 ligand in murine systemic lupus erythematosus. J Immunol 168: 2046–2053. 11823542 10.4049/jimmunol.168.4.2046

[17] Kawai T, Andrews D, Colvin RB, Sachs DH, Cosimi AB (2000) Thromboembolic complications after treatment with monoclonal antibody against CD40 ligand. Nat Med 6: 114. 10.1038/72162 10655073

[18] Robles-Carrillo L, Meyer T, Hatfield M, Desai H, Davila M, et al (2010) Anti-CD40L immune complexes potently activate platelets in vitro and cause thrombosis in FCGR2A transgenic mice. J Immunol 185: 1577–1583. 20585032 10.4049/jimmunol.0903888

[19] Qin H, Wilson CA, Lee SJ, Zhao X, Benveniste EN (2005) LPS induces CD40 gene expression through the activation of NF-kappaB and STAT-1alpha in macrophages and microglia. Blood 106: 3114–3122. 16020513 10.1182/blood-2005-02-0759 PMC1895321

[20] Vowinkel T, Wood KC, Stokes KY, Russell J, Krieglstein CF, et al (2006) Differential expression and regulation of murine CD40 in regional vascular beds. Am J Physiol Heart Circ Physiol 290: H631–639. 16172156 10.1152/ajpheart.00733.2005

[21] Layzer JM, McCaffrey AP, Tanner AK, Huang Z, Kay MA, et al (2004) In vivo activity of nuclease-resistant siRNAs. RNA 10: 766–771. 15100431 10.1261/rna.5239604 PMC1370566

[22] Choung S, Kim YJ, Kim S, Park HO, Choi YC (2006) Chemical modification of siRNAs to improve serum stability without loss of efficacy. Biochem Biophys Res Commun 342: 919–927. 16598842 10.1016/j.bbrc.2006.02.049

[23] Allerson CR, Sioufi N, Jarres R, Prakash TP, Naik N, et al (2005) Fully 2′-modified oligonucleotide duplexes with improved in vitro potency and stability compared to unmodified small interfering RNA. J Med Chem 48: 901–904. 15715458 10.1021/jm049167j

[24] Corey DR (2007) Chemical modification: the key to clinical application of RNA interference? J Clin Invest 117: 3615–3622. 18060019 10.1172/JCI33483 PMC2096450

[25] Soutschek J, Akinc A, Bramlage B, Charisse K, Constien R, et al (2004) Therapeutic silencing of an endogenous gene by systemic administration of modified siRNAs. Nature 432: 173–178. 15538359 10.1038/nature03121

[26] Ripoll E, Pluvinet R, Torras J, Olivar R, Vidal A, et al (2011) In vivo therapeutic efficacy of intra-renal CD40 silencing in a model of humoral acute rejection. Gene Ther 18: 945–952. 21472009 10.1038/gt.2011.39

[27] Pluvinet R, Olivar R, Krupinski J, Herrero-Fresneda I, Luque A, et al (2008) CD40: an upstream master switch for endothelial cell activation uncovered by RNAi-coupled transcriptional profiling. Blood 112: 3624–3637. 18669876 10.1182/blood-2008-03-143305

[28] Espeli M, Bokers S, Giannico G, Dickinson HA, Bardsley V, et al (2011) Local renal autoantibody production in lupus nephritis. J Am Soc Nephrol 22: 296–305. 21088295 10.1681/ASN.2010050515 PMC3029902

[29] Peters AL, Stunz LL, Bishop GA (2009) CD40 and autoimmunity: the dark side of a great activator. Semin Immunol 21: 293–300. 19595612 10.1016/j.smim.2009.05.012 PMC2753170

[30] Klinman DM (1990) Polyclonal B cell activation in lupus-prone mice precedes and predicts the development of autoimmune disease. J Clin Invest 86: 1249–1254. 2212009 10.1172/JCI114831 PMC296855

[31] Odendahl M, Jacobi A, Hansen A, Feist E, Hiepe F, et al (2000) Disturbed peripheral B lymphocyte homeostasis in systemic lupus erythematosus. J Immunol 165: 5970–5979. 11067960 10.4049/jimmunol.165.10.5970

[32] Shimomura Y, Mizoguchi E, Sugimoto K, Kibe R, Benno Y, et al (2008) Regulatory role of B-1 B cells in chronic colitis. Int Immunol 20: 729–737. 18375938 10.1093/intimm/dxn031

[33] Park YB, Lee SK, Kim DS, Lee J, Lee CH, et al (1998) Elevated interleukin-10 levels correlated with disease activity in systemic lupus erythematosus. Clin Exp Rheumatol 16: 283–288. 9631750

[34] Llorente L, Zou W, Levy Y, Richaud-Patin Y, Wijdenes J, et al (1995) Role of interleukin 10 in the B lymphocyte hyperactivity and autoantibody production of human systemic lupus erythematosus. J Exp Med 181: 839–844. 7869046 10.1084/jem.181.3.839 PMC2191898

[35] Mosser DM, Zhang X (2008) Interleukin-10: new perspectives on an old cytokine. Immunol Rev 226: 205–218. 19161426 10.1111/j.1600-065X.2008.00706.x PMC2724982

[36] Cassese G, Lindenau S, de Boer B, Arce S, Hauser A, et al (2001) Inflamed kidneys of NZB/W mice are a major site for the homeostasis of plasma cells. Eur J Immunol 31: 2726–2732. 11536171 10.1002/1521-4141(200109)31:9<2726::aid-immu2726>3.0.co;2-h

[37] Starke C, Frey S, Wellmann U, Urbonaviciute V, Herrmann M, et al (2011) High frequency of autoantibody-secreting cells and long-lived plasma cells within inflamed kidneys of NZB/W F1 lupus mice. Eur J Immunol 41: 2107–2112. 21484784 10.1002/eji.201041315

[38] Sekine H, Ruiz P, Gilkeson GS, Tomlinson S (2011) The dual role of complement in the progression of renal disease in NZB/W F(1) mice and alternative pathway inhibition. Mol Immunol 49: 317–323. 22000720 10.1016/j.molimm.2011.09.015

[39] Manderson AP, Botto M, Walport MJ (2004) The role of complement in the development of systemic lupus erythematosus. Annu Rev Immunol 22: 431–456. 15032584 10.1146/annurev.immunol.22.012703.104549

[40] Tang S, Zhou W, Sheerin NS, Vaughan RW, Sacks SH (1999) Contribution of renal secreted complement C3 to the circulating pool in humans. J Immunol 162: 4336–4341. 10201966

[41] Sledz CA, Holko M, de Veer MJ, Silverman RH, Williams BR (2003) Activation of the interferon system by short-interfering RNAs. Nat Cell Biol 5: 834–839. 12942087 10.1038/ncb1038

[42] Alexopoulou L, Holt AC, Medzhitov R, Flavell RA (2001) Recognition of double-stranded RNA and activation of NF-kappaB by Toll-like receptor 3. Nature 413: 732–738. 11607032 10.1038/35099560

[43] Reynolds A, Anderson EM, Vermeulen A, Fedorov Y, Robinson K, et al (2006) Induction of the interferon response by siRNA is cell type- and duplex length-dependent. RNA 12: 988–993. 16611941 10.1261/rna.2340906 PMC1464853

