# Correction: CD40 Gene Silencing Reduces the Progression of Experimental Lupus Nephritis Modulating Local Milieu and Systemic Mechanisms

**DOI:** 10.1371/annotation/8494360d-8ab1-4575-8a01-fb288ffde976

**Published:** 2013-08-20

**Authors:** Èlia Ripoll, Ana Merino, Montse Goma, Josep M. Aran, Nuria Bolaños, Laura de Ramon, Immaculada Herrero-Fresneda, Oriol Bestard, Josep M. Cruzado, Josep M. Grinyó, Juan Torras

The author list for the article should be corrected to list Dr Herrero-Fresneda as third author and Dr Goma as fifth author, the revised author list should thus read as follows:

Èlia Ripoll, Ana Merino, Immaculada Herrero-Fresneda, Josep M. Aran, Montse Goma, Nuria Bolaños, Laura de Ramon, Oriol Bestard, Josep M. Cruzado, Josep M. Grinyó, Juan Torras.

The details of authors' contributions should also be corrected to reflect the following contributions:

Conceived and designed the experiments: ER, JMA, JMG, IHF, JT. Performed the experiments: ER AM MG NB IHF. Analyzed the data: ER OB JMC JMG IHF JT. Contributed reagents/materials/analysis tools: MG NB LdR IHF. Wrote the paper: ER IHF JT. 

